# Supplementary figures and images for: Human Fallopian Tube-Derived Organoids with TP53 and RAD51D Mutations Recapitulate an Early Stage High-Grade Serous Ovarian Cancer Phenotype In Vitro
Source: Int J Mol Sci. 2024 Jan 10;25(2):886. doi: 10.3390/ijms25020886 (PMC10815309; doi:10.3390/ijms25020886)

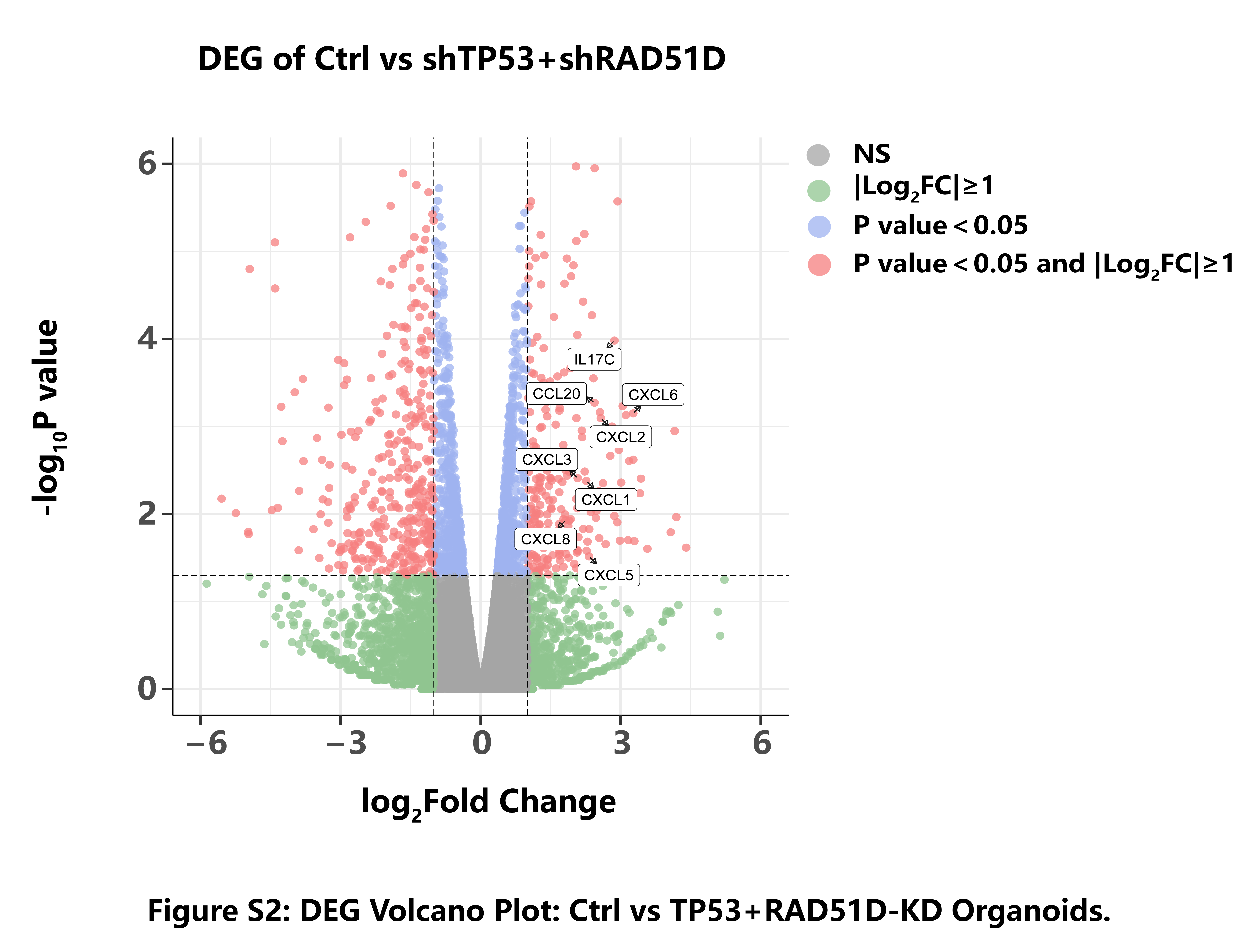

Supplement: Supplementary file 1 [file ijms-25-00886-s001.zip › supplementary figure S2.tif]
